# Repression of Transcriptional Activity of Forkhead Box O1 by Histone Deacetylase Inhibitors Ameliorates Hyperglycemia in Type 2 Diabetic Rats

**DOI:** 10.3390/ijms19113539

**Published:** 2018-11-09

**Authors:** Hyun Min Cho, Young Mi Seok, Hae Ahm Lee, Minji Song, InKyeom Kim

**Affiliations:** 1 Department of Pharmacology, School of Medicine, Kyungpook National University, Daegu 41944, Korea; hmcho.7998@gmail.com (H.M.C.); hae.ahm.lee@gmail.com (H.A.L.); smj2313@hanmail.net (M.S.); 2 Cardiovascular Research Institute, School of Medicine, Kyungpook National University, Daegu 41944, Korea; abbamaria@hanmail.net; 3 National Development Institute of Korean Medicine, Gyeongsan, Gyeongbuk 38540, Korea; 4 Department of Medical Zoology, Kyung Hee University School of Medicine, Seoul 02447, Korea

**Keywords:** type 2 diabetes mellitus, histone deacetylase, histone deacetylase inhibitors, FoxO1 acetylation, transcriptional regulation

## Abstract

Type 2 diabetes mellitus (T2DM) is a chronic disease manifested by hyperglycemia. It is essential to effectively control hyperglycemia to prevent complications of T2DM. Here, we hypothesize that repression of transcriptional activity of forkhead box O1 (FoxO1) via histone deacetylase inhibitors (HDACi) ameliorates hyperglycemia in T2DM rats. Methods: Male Long-Evans Tokushima Otsuka (LETO) and Otsuka Long-Evans Tokushima Fatty (OLETF) rats aged 14 weeks were administered sodium valproate (VPA, 0.71% *w*/*v*) dissolved in water for 20 weeks. Electrophoretic mobility shift assay (EMSA) and luciferase assay were performed for elucidation of transcriptional regulation through acetylation of FoxO1 by HDACi. Results: VPA attenuated blood glucose levels in accordance with a decrease in the expression of gluconeogenic genes in hyperglycemic OLETF rats. It has been shown that HDAC class I-specific and HDAC class IIa-specific inhibitors, as well as pan-HDAC inhibitors decrease FoxO1 enrichment at the *cis*-element of target gene promoters. Mutations in FoxO1 prevent its acetylation, thereby increasing its transcriptional activity. HDAC3 and HDAC4 interact with FoxO1, and knockdown of HDAC3, HDAC4, or their combination increases FoxO1 acetylation, thereby decreasing the expression of gluconeogenic genes. Conclusions: These results indicate that HDACi attenuates the transcriptional activity of FoxO1 by impeding deacetylation, thereby ameliorating hyperglycemia in T2DM rats.

## 1. Introduction

It is estimated that 371 million people have diabetes mellitus, and 1.5 million Americans are newly diagnosed with diabetes every year [1]. Most patients with diabetes also suffer from long-term complications, including retinopathy, nephropathy, peripheral neuropathy, and cardiovascular disease [2]. The economic burden of diabetes mellitus exceeded $327 billion in total costs of diagnosed diabetes in the United States in 2017 and $237 billion in direct medical costs in 2017 (http://www.diabetes.org/diabetes-basics/statistics/, accessed on 22 March 2018).

There are two primary forms of diabetes mellitus. Type I diabetes mellitus, which is caused by a deficiency in insulin secretion, accounts for 5–10% of people with diabetes. Type II diabetes mellitus (T2DM), which is caused by increased insulin resistance and decreased insulin secretion, accounts for 90–95% of people with diabetes [3]. Glucose homeostasis is controlled by insulin and glucagon, which are opposing pancreatic hormones. During starvation, glucagon promotes gluconeogenesis by activating peroxisome proliferator-activated receptor gamma coactivator 1 alpha (PGC1α), which interacts with forkhead box protein O1 (FoxO1) and increases the synthesis of glucose 6-phosphatase (G6P) and phosphoenolpyruvate carboxykinase (PCK1), consequently increasing gluconeogenesis in the liver [4]. In contrast, insulin decreases hepatic glucose production during states of nutrient abundance by activating the AKT Ser/Thr kinase and subsequent phosphorylation of FoxO1, thereby inhibiting nuclear translocation and increasing glucose uptake in peripheral tissues [4,5]. Dysfunction of these hormones leads to hyperglycemia (high glucose levels), which is a major symptom of diabetes mellitus.

Histone deacetylases (HDACs) are grouped into four classes according to sequence similarities among their amino acid and structural features, including class I HDACs (1, 2, 3, and 8), class IIa HDACs (4, 5, 7, and 9), class IIb HDACs (6 and 10), and class IV HDACs (11) [6]. HDACs deacetylate non-histone proteins and were originally recognized as important enzymes involved in epigenetic gene silencing via histone deacetylation. HDAC-mediated target protein deacetylation functions as a negative regulator [7,8,9,10]. In addition, HDACs facilitate the transcriptional activity of mineralocorticoid receptor (MR), as shown in our previous study [11]. Dysregulation of acetylation levels is linked to many pathological diseases, and inhibitors of HDACs exhibit anti-fibrotic, anti-inflammatory, anti-hypertrophic, and anti-hypertensive properties [12,13].

Recently, a role for HDACs in diabetes mellitus has emerged. Two pan-HDAC inhibitors, trichostatin A (TSA) and sodium butyrate, have been shown to increase histone H4 acetylation, which stimulates the transcription of insulin-related genes [14]. Depletion of HDAC2 via RNA interference or TSA partially restores insulin signaling by acetylating the insulin receptor (IR) substrate 1 [15]. MS275, a class I specific HDAC inhibitor, ameliorates metabolic dysfunction and stimulates mitochondrial function by inducing PGC1α in a diet-induced obese mouse model [16]. In addition to TSA, suberoylanilide hydroxamic acid (SAHA), prevents cytokine-induced beta cell death and improves beta cell function, which is thought to downregulate nuclear factor kappa B (NFκB) activity [17]. Although various HDAC inhibitors are used to treat diabetes mellitus, the molecular mechanisms are still unclear. We hypothesized that repression of transcriptional activity of forkhead box O1 (FoxO1) via histone deacetylase inhibitors (HDACi) ameliorates hyperglycemia in type 2 diabetic rats.

## 2. Results

### 2.1. Administration of Sodium Valproate (VPA) Ameliorates Blood Glucose Levels in Otsuka Long-Evans Tokushima Fatty (OLETF) Rats

Otsuka Long-Evans Tokushima Fatty (OLETF) rats are rats from which are one of the obesity and type 2 diabetes mellitus models were derived, from a spontaneous obesity in an out-bred colony of Long Evans rats. OLETF and a control Long-Evans Tokushima Otsuka (LETO) lines were then developed by selective breeding. OLETF rats were initially studied primarily as a model of late onset type 2 diabetes, as older OLETF rats were not only obese but also hyperglycemic and insulin resistant [18].

**Figure 1:**
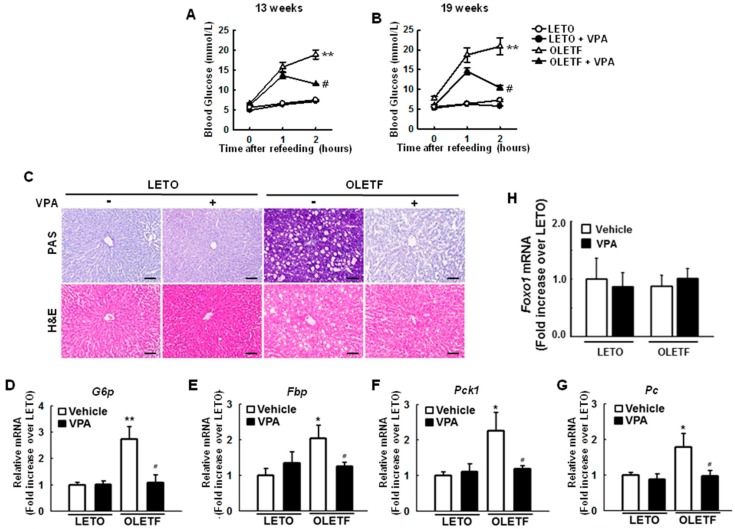
Sodium valproate (VPA) reduces gluconeogenesis in type 2 diabetic rats. (**A**,**B**) Blood glucose levels increased 1 and 2 h after re-feeding in Otsuka Long-Evans Tokushima Fatty (OLETF) rats, an effect that was ameliorated by VPA administration; (**C**) Glycogen accumulation was analyzed by periodic acid–Schiff (PAS) staining of liver tissues. Representative images show the effect of VPA administration on glycogen accumulation in liver tissues (*n* = 6, scale bar = 50 μm); Expression of gluconeogenic genes, such as glucose 6-phosphatase ((**D**), *G6p*), fructose-1, 6-bisphosphatase (**E**, *Fbp*), phosphoenolpyruvate carboxykinase ((**F**), *Pck1*); and pyruvate carboxylase ((**G**), *Pc*) was quantified by reverse transcription-quantitative PCR. VPA administration decreased the expression of gluconeogenic genes in OLETF rats. *Foxo1* mRNA was not significantly different between Long-Evans Tokushima Otsuka (LETO) and OLETF rats regardless of VPA treatment (**H**). The graphs show the mean ± standard error of mean (SEM) of six independent experiments. (* *p* < 0.05, ** *p* < 0.01 vs. Long-Evans Tokushima Otsuka (LETO) rats; ^#^ *p* < 0.05 vs. OLETF vehicle).

To investigate the effects of HDAC inhibition on glucose metabolism, we administered VPA, a pan-HDAC inhibitor, to 14-week old LETO rats or OLETF rats. VPA (0.71% *w*/*v*) was dissolved in drinking water and administered for 20 weeks. Blood glucose levels increased 1 and 2 h after re-feeding, an effect that was significantly ameliorated by VPA administration for 13 weeks, as shown in Figure 1A, and 19 weeks, as shown in Figure 1B, in OLETF rats. Periodic acid-Schiff (PAS) staining showed that glycogen accumulation was greater in OLETF rats than that in LETO rats, an effect that was overcome with VPA administration, as shown in Figure 1C. To determine how VPA reduces blood glucose levels, we investigated the expression of gluconeogenic genes in the liver of rats. The expression levels of gluconeogenic genes such as glucose 6-phosphatase (*G6p*), fructose-1,6-bisphosphatase (*Fbp*), phosphoenolpyruvate carboxykinase (*Pck1*), and pyruvate carboxylase (*Pc*) were more than 2-fold higher (*p* < 0.05) in OLETF rats than those in LETO rats; these phenomena were attenuated in rats administered VPA for 20 weeks, as shown in Figure 1D–G. Interestingly, there were no significant differences in the expression of *Foxo1* mRNA between LETO and OLETF rats regardless of VPA treatment, as shown in Figure 1H.

### 2.2. High Glucose Conditions Induce the Expression of G6P and PCK1 Genes in HepG2 Cells

**Figure 2:**
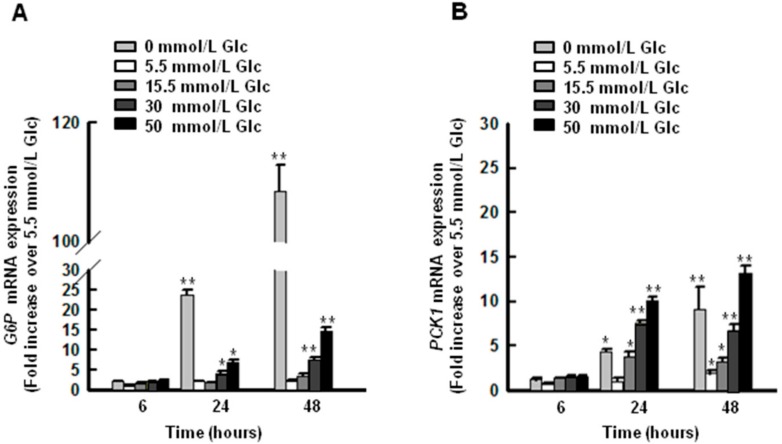
*G6P* and *PCK1* gene expression increase with increasing glucose concentrations in HepG2 cells. Expression of *G6P* (**A**) and *PCK1* (**B**) increased under hyperglycemic conditions (30 and 50 mmol/L glucose in media). The graphs show the mean ± SEM of three independent experiments. (* *p* < 0.05, ** *p* < 0.01 vs. 5.5 mmol/L at 24 h).

To develop a hyperglycemic model in hepatic cells, HepG2 cells were exposed to increasing doses of glucose (0, 5.5, 15.5, 30, and 50 mmol/L) for 6 to 48 h, and the expression of key proteins related to gluconeogenesis was evaluated. *G6P* and *PCK1* mRNAs increased in HepG2 cells with all glucose concentrations tested, as shown in Figure 2A,B. *G6P* mRNA was approximately 3-fold higher after 24 h in cells treated with 30 and 50 mmol/L glucose than that in cells treated with 5.5 mmol/L glucose. For someone without diabetes, fasting blood glucose on awakening should be under 100 mg/dL (≈5.5 mmol/L, molecular weight of glucose: 180.16). Thus, the data was normalized to 5.5 mmol/L, which concentration mimics normal blood glucose. *PCK1* mRNA was approximately 7-fold higher after 24 h in cells treated with 30 and 50 mmol/L glucose than that in cells treated with 5.5 mmol/L glucose. Both *G6P* and *PCK1* mRNAs were significantly higher after 24 h in cells receiving no glucose than those in cells receiving 5.5 mmol/L glucose for 6 h. These data suggest that high concentrations of glucose (30 and 50 mmol/L) induce gluconeogenesis in hepatocyte cells.

### 2.3. FoxO1 Enrichment at Target Genes Is Increased under Hyperglycemic Conditions and Is Disturbed by HDAC Inhibitors in HepG2 Cells

**Figure 3:**
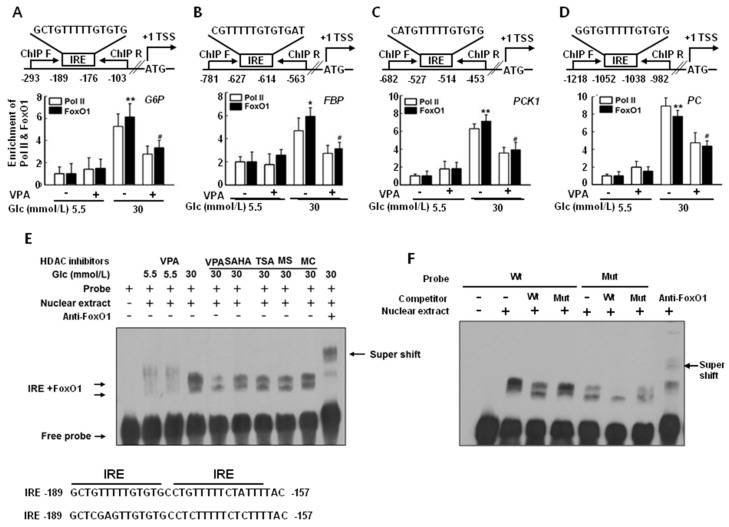
Histone deacetylase (HDAC) inhibitors decrease recruitment of forkhead box O1 (FoxO1) to the *cis*-element of gluconeogenic genes. Recruitment of FoxO1 and polymerase II to glucose 6-phosphatase (*G6P*, (**A**)); fructose-1, 6-bisphosphatase (*FBP*, (**B**)); phosphoenolpyruvate carboxykinase (*PCK1*, (**C**)); and pyruvate carboxylase (*PC*, (**D**)) increased under hyperglycemic conditions (30 mmol/L glucose), an effect that was inhibited by administration of VPA (10 mmol/L). The graphs show the mean ± SEM of three independent experiments (* *p* < 0.05, ** *p* < 0.01 vs. vehicle in 5.5 mmol/L glucose; ^#^ *p* < 0.05 vs. vehicle 30 mmol/L glucose); (**E**) Electrophoretic mobility shift assay (EMSA) was performed using oligonucleotides of the insulin response element (IRE) as a probe. The probe formed complexes (arrow: →) with nuclear extract; these were decreased by VPA (10 mmol/L), suberoylanilide hydroxamic acid (SAHA, 10 μmol/L), trichostatin A (TSA, 1 μmol/L), MS275 (100 μmol/L), and MC1568 (10 μmol/L). HDAC inhibitors were treated for 24 h; (**F**) A 100-fold excess of the competitor, but not mutated probe, competed with the complex formation. Complexes that formed with the normal, but not mutated probes, were super-shifted by FoxO1 antibodies (arrow: ←).

To determine whether FoxO1, a transcription factor, is recruited to the promoter region of gluconeogenic genes under hyperglycemic conditions (30 mmol/L glucose) and whether VPA decreases that recruitment, we performed chromatin immunoprecipitation (ChIP) analyses using FoxO1 antibodies and ChIP primers. Enrichment of FoxO1 and RNA polymerase II (Pol II) at the *G6P*, *FBP*, *PCK1*, and *PC* promoter regions was higher under hyperglycemic conditions (30 mmol/L glucose) than that under euglycemic conditions (5.5 mmol/L glucose) without VPA treatment, as shown in Figure 3A–D. In addition, results show that VPA treatment reduced FoxO1 and Pol II enrichment, resulting in reduced gene expression. Therefore, increments in mRNA expression correlating with FoxO1 binding affinity at the *G6P*, *FBP*, *PCK1*, and *PC* promoter regions are dependent on glucose concentration. In silico analysis using the Transcription Element Search System (http://gene-regulation.com/pub/programs/alibaba2/index.html; accessed on 7 November 2018) suggested the presence of a putative binding site (−189/−157) in the *G6P* promoter for human FoxO1, as shown in the lower part of Figure 3E. Direct evidence for the interaction between the insulin response element (IRE), a consensus *cis*-element of the promoter region, and FoxO1 was validated by an electrophoretic mobility shift assay using HepG2 cell nuclear extracts and an oligonucleotide probe (IRE: 5′-GCTGTTTTTGTGTGCCTGTTTTTCTATTTTAC-3′; IRE-mut: 5′ GCTCGAGTTGTGTGCCTCTTTTTCTCTTTTAC-3′). A weak interaction between FoxO1 and the IRE probe was noted under euglycemic conditions, and the interaction was not affected by VPA treatment. In contrast, a strong interaction between FoxO1 and the IRE probe was noted under hyperglycemic conditions, an effect that was reduced by HDAC inhibitors, including VPA, SAHA, TSA, MS275, and MC1568, as shown in Figure 3E. A mutant IRE probe showed a weak interaction with FoxO1, as shown in Figure 3F.

These results suggest that interactions between FoxO1 and the IRE are stronger under hyperglycemic conditions than those under euglycemic conditions, and HDAC inhibitors impede these interactions.

### 2.4. Transcriptional Activity of FoxO1 Is Attenuated by Acetylation

**Figure 4:**
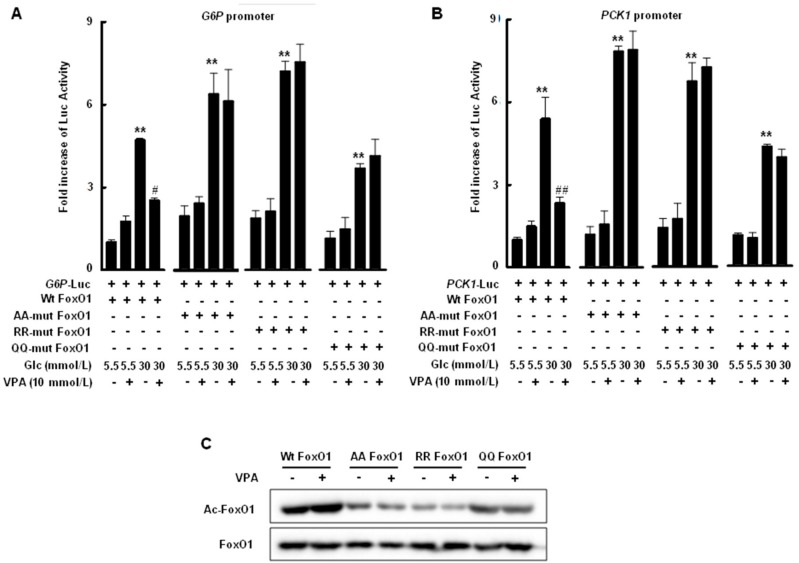
VPA reduced transcriptional activity of FoxO1 via acetylation. HepG2 cells were co-transfected with expression vectors for wild-type (wt) FoxO1, AA-mutant FoxO1, RR-mutant FoxO1, or QQ-mutant FoxO1, as well as luciferase vectors conjugated with *G6P* promoter (**A**) or *PCK1* promoter (**B**). VPA administration decreased hyperglycemia-induced promoter activity in HepG2 cells transfected with wild-type FoxO1, but not mutant FoxO1. The graphs show the mean ± SEM of three independent experiments (** *p* < 0.01 vs. wt FoxO1 in 5.5 mmol/L glucose; ^#^ *p* < 0.05, ^##^ *p* < 0.01 vs. wt FoxO1 in 30 mmol/L glucose); (**C**) VPA administration under hyperglycemic conditions resulted in increased FoxO1 acetylation in HepG2 cells transfected with wt FoxO1, but not mutant FoxO1.

A luciferase assay was used to evaluate promoter activity in response to hyperglycemic conditions and the acetylation state of FoxO1 protein. The human *G6P* promoter (−1879/+95) or human *PCK1* promoter (−1875/+120) was fused with the pGL3-Luc vector and induced by hyperglycemic conditions. Promoter activity was significantly decreased by VPA treatment only when HepG2 cells were transfected with wild-type FoxO1 under hyperglycemic conditions, as shown in Figure 4A,B; K242, K245, and K262 in the DNA binding domain of FoxO1 were substituted with alanine (AA mutant FoxO1), arginine (RR mutant FoxO1), or glutamine (QQ mutant FoxO1). *G6P* promoter-fused pGL3-Luc and *PCK1* promoter-fused pGL3-Luc in HepG2 cells transfected with each mutant FoxO1 were induced under hyperglycemic conditions; however, these effects were not reduced by VPA treatment. Mutations in FoxO1 resulted in lower FoxO1 acetylation, despite VPA treatment, than that observed for wild-type FoxO1, as shown in Figure 4C. 

### 2.5. Hyperglycemic Conditions Induce Interactions between FoxO1 and HDAC3, as Well as FoxO1 and HDAC4, Which Regulate the Transcriptional Activity of FoxO1

**Figure 5:**
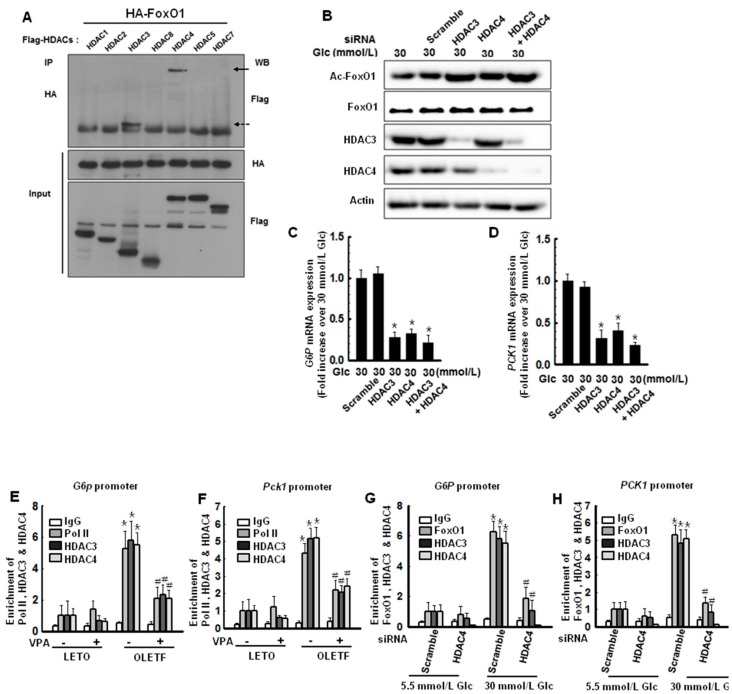
Histone deacetylase 3 (HDAC3) and HDAC4 are responsible for FoxO1 deacetylation. (**A**) HepG2 cells were co-transfected with hemagglutinin (HA)-FoxO1 and either Flag-HDAC1, Flag-HDAC2, Flag-HDAC3, Flag-HDAC4, Flag-HDAC5, or Flag-HDAC7. FoxO1 was precipitated by anti-HA antibodies and FoxO1-interacting HDACs were detected by western blotting with an anti-Flag antibody; (**B**) HepG2 cells were transfected with HDAC3 siRNA, HDAC4 siRNA, or a combination for 48 h, resulting in increased FoxO1 acetylation. Knockdown of HDAC3, HDAC4, or a combination significantly attenuated the expression of *G6P* (**C**) and *PCK1* (**D**); The graphs show the mean ± SEM of three independent experiments (* *p* < 0.05 vs. scrambled). Recruitment of Pol II, HDAC3 or HDAC4 on *G6p* promoter (**E**) or *Pck1* promoter (**F**) was decreased when VPA was administered for 20 weeks in OLETF rats. The graphs show the mean ± SEM of six independent experiments (* *p* < 0.05 vs. LETO vehicle; ^#^ *p* < 0.05 vs. OLETF vehicle); Recruitment of FoxO1, HDAC3 or HDAC4 on *G6P* promoter (**G**) or *PCK1* promoter (**H**) was decreased when HDAC4 was depleted by HDAC4 siRNA in HepG2 cells. The graphs show the mean ± SEM of three independent experiments (* *p* < 0.05 vs. 5.5 mM Glc scramble; ^#^ *p* < 0.05 vs. 30 mmol/L scramble). IP: immunoprecipitation.

Mihaylova et al. [19] showed that loss of class IIa HDACs in murine liver results in inhibition of FoxO target genes and reduced blood glucose levels. To identify which HDAC interacts with FoxO1, hemagglutinin (HA)-tagged FoxO1 and flag-tagged HDAC1, 2, 3, 8, 4, 5, or 7 were co-transfected into HepG2 cells and co-immunoprecipitated (co-IP) to investigate the interactions between FoxO1 and class I and class IIa HDACs. Under hyperglycemic conditions (30 mmol/L glucose), HDAC3 and HDAC4 interacted with FoxO1, as shown in Figure 5A. We further analyzed FoxO1 acetylation, which reduces its DNA binding affinity. Interactions between FoxO1 and the IRE were reduced by treatment with VPA, MS275, and MC1568, as shown in Figure 3E. Therefore, we speculated that both HDAC3 and HDAC4 inhibitors have a role in FoxO1 deacetylation and regulation of FoxO1 transcriptional activity. To evaluate this hypothesis, we performed knockdown experiments with HDAC3, HDAC4, or both. As expected, knockdown of HDAC3, HDAC4, or both resulted in more FoxO1 acetylation than that observed for the scrambled control, as shown in Figure 5B. Expression of the target genes, *G6P* and *PCK1*, was significantly lower in cells with reduced expression of HDAC3, HDAC4, or both, as shown in Figure 5C,D.

### 2.6. HDAC Inhibitors Decrease the Expression of G6P and PCK1 Genes under Hyperglycemic Conditions

**Figure 6:**
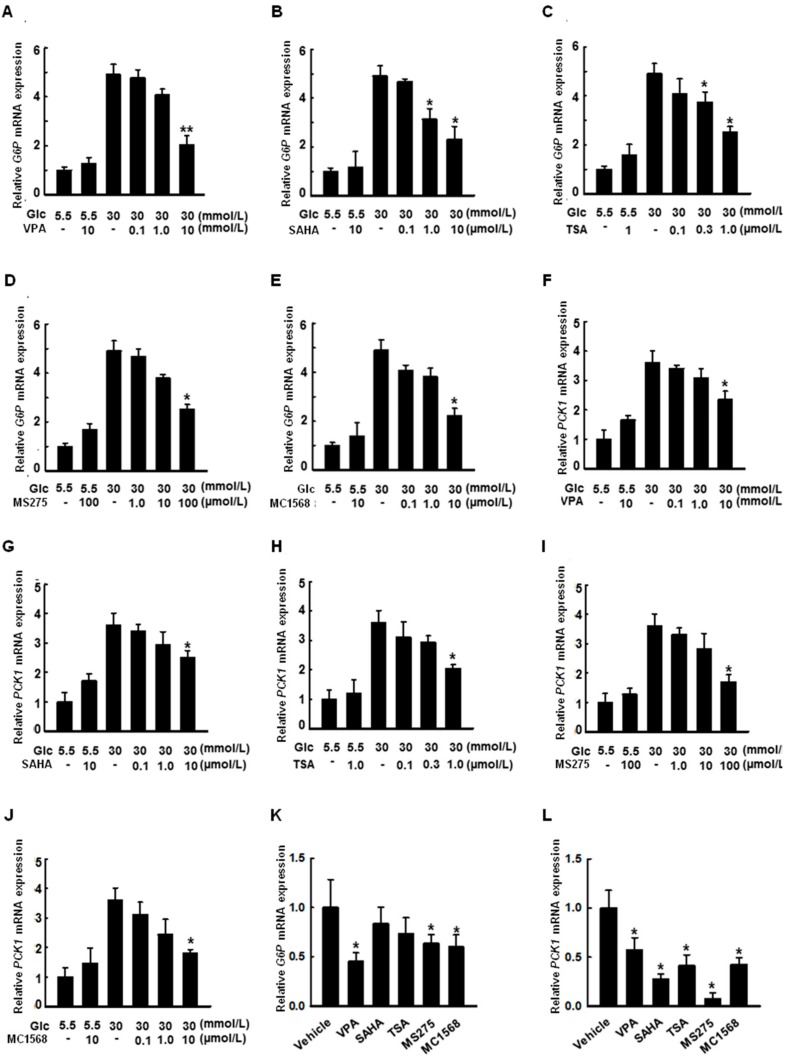
Histone deacetylase (HDAC) inhibitors reduce the expression of *G6P* and *PCK1* in HepG2 cells. Cells were treated with VPA ((**A**,**F**), 0.1, 1.0, and 10 mmol/L); SAHA ((**B**,**G**), 0.1, 1.0, and 10 µmol/L); TSA (**C**, **H**, 0.1, 0.3, and 1.0 µmol/L); MS275 ((**D**,**I**), 1.0, 10, and 100 µmol/L); and MC1568 ((**E**,**J**), 0.1, 1.0, and 10 µmol/L) for 48 h under hyperglycemic conditions. Expression of *G6P* or *PCK1* was quantified by RT-qPCR. Cultivation under hyperglycemic conditions increased the expression of *G6P* or *PCK1*, an effect that was attenuated by treatment with the pan-HDAC inhibitors VPA, SAHA, and TSA, as well as the HDAC class I-specific inhibitor, MS275, and the HDAC class IIa-specific inhibitor, MC1568. The graphs show the mean ± SEM of three independent experiments (* *p* < 0.05, ** *p* < 0.01 vs. vehicle in 30 mmol/L glucose). HDAC inhibitors (VPA, 10 mmol/L; SAHA, 10 µmol/L; TSA, 1.0 µmol/L; MS275, 100 µmol/L; MC1568, 10 µmol/L, for 24 h) also decreased the expression of *G6P* or *PCK1* in the absence of glucose (**K**,**L**). The graphs show the mean ± SEM of three independent experiments (* *p* < 0.05 vs. vehicle in 0 mmol/L glucose).

Cells were treated with VPA (Figure 6A,F, 0.1, 1.0, and 10 mmol/L), SAHA (Figure 6B,G, 0.1, 1.0, and 10 µmol/L), TSA (Figure 6C,H, 0.1, 0.3, and 1.0 µmol/L), MS275 (Figure 6D,I, 1.0, 10, and 100 µmol/L), and MC1568 (Figure 6E,J, 0.1, 1.0, and 10 µmol/L) for 48 h under hyperglycemic conditions. *G6P* and *PCK1* expression was increased under hyperglycemic conditions, an effect that was decreased by treatment with the pan-HDAC inhibitors VPA, SAHA, and TSA, as well as the HDAC class I-specific inhibitor, MS275, and the HDAC class II a-specific inhibitor, MC1568.

### 2.7. VPA Administration Decreases FoxO1 Enrichment at the Target Genes in Type 2 Diabetic Rats

**Figure 7:**
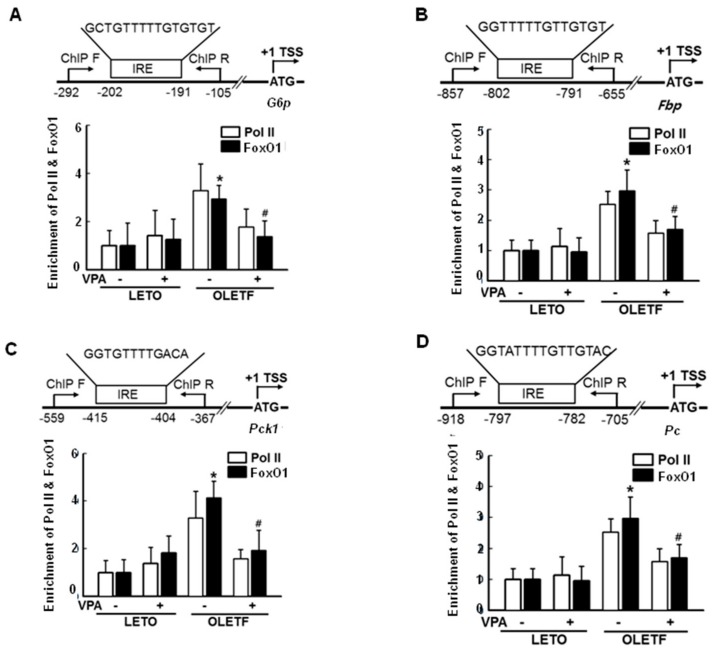
VPA reduces FoxO1 recruitment at target genes in type 2 diabetic rats. Recruitment of FoxO1 and polymerase II to *G6p* (**A**); *Fbp* (**B**); *Pck1* (**C**); or *Pc* (**D**) was higher in OLETF rats than that in LETO rats, an effect that was reduced by VPA administration for 20 weeks. The graphs show the mean ± SEM of six independent experiments (* *p* < 0.05 vs. vehicle LETO; ^#^ *p* < 0.05 vs. vehicle OLETF).

To determine whether FoxO1 is recruited to the promoter of gluconeogenic genes in diabetic OLETF rats and whether VPA decreases this recruitment, we performed a ChIP analysis with FoxO1 antibodies; enrichment of FoxO1 and Pol II at the *G6p*, *Fbp*, *Pck1*, and *Pc* promoter regions, as shown in Figure 7A–D, was higher in OLETF rats than in LETO rats. Further, FoxO1 and Pol II enrichment was reduced in the presence of VPA, resulting in decreased gene expression. Therefore, increments in mRNA expression correlated with the binding affinity of FoxO1 at the *G6p*, *Fbp*, *Pck1*, and *Pc* promoter, and VPA regulated the binding activity in OLETF rats.

### 2.8. VPA Administration Increases FoxO1 Acetylation in Type 2 Diabetic Rats

Interestingly, there were no significant differences in the expression of FoxO1 protein as *Foxo1* mRNA, as shown in Figure 1H, between LETO and OLETF rats regardless of VPA treatment.

**Figure 8:**
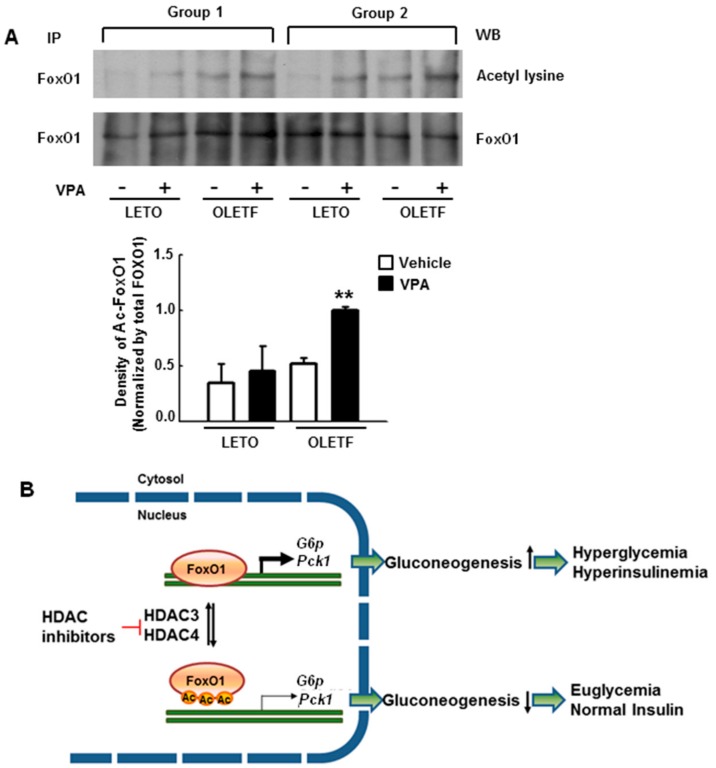
VPA maintains acetylation of FoxO1 and attenuates hyperglycemia in type 2 diabetic rats. FoxO1 protein was not significantly different between LETO and OLETF rats regardless of VPA treatment (**A**); FoxO1 acetylation was analyzed by western blot with anti-acetyl-lysine antibodies after immunoprecipitation (IP) with anti-FoxO1 antibodies. A representative immunoblot showing that FoxO1 acetylation is increased by VPA administration is presented. Data show the mean ± SEM of five independent experiments (** *p* < 0.01 vs. vehicle OLETF); (**B**) A summary of the involvement of histone deacetylase (HDAC) inhibitors in gluconeogenesis is shown. HDAC inhibitors increased acetylation of FoxO1 by inhibiting HDAC3 and HDAC4, which interfered with the DNA binding activity of FoxO1.

To investigate the effects of HDAC inhibition on FoxO1 acetylation, western blots were performed after immunoprecipitation (IP) with anti-FoxO1 antibodies in the liver tissues. VPA treatment had little effect on FoxO1 acetylation in LETO rats, whereas it led to a significant increase in FoxO1 acetylation in OLETF rats, as shown in Figure 8A. These results suggest that FoxO1 up-regulates gluconeogenic genes in diabetic rats, resulting in severe hyperglycemia. However, HDAC3 and HDAC4 inhibition attenuates hyperglycemia via FoxO1 acetylation, as shown in Figure 8B.

## 3. Discussion

The present study demonstrates that repression of transcriptional activity of FoxO1 by HDAC inhibitors ameliorates hyperglycemia in type 2 diabetic rats. The decrease in blood glucose levels coincided with a decrease in the expression of gluconeogenic genes via FoxO1 acetylation in hyperglycemic OLETF rats administered VPA. FoxO1 enrichment at the promoter sites of gluconeogenic genes increased under hyperglycemic conditions, an effect that was disturbed by HDAC inhibitors, including VPA, in HepG2 cells. Knockdown of HDAC3 and HDAC4 increased FoxO1 acetylation, thereby leading to downregulation of gluconeogenic genes. 

The FoxO subfamily is ubiquitously expressed and highly conserved [20]. Especially, FoxO1 plays an important role in regulating the insulin response in the liver; insulin sensitivity is increased in the liver via AKT phosphorylation when FoxO1 is constitutively expressed [21]. In the present study, we demonstrated that VPA administration increases FoxO1 acetylation, as shown in Figure 8A, and decreases the expression of gluconeogenic genes in OLETF rats, as shown in Figure 1D–G. Further, HDAC inhibitors decreased the expression of gluconeogenic genes in HepG2 cells, a shown in Figure 6. FoxO1 is acetylated at K242, K245, K248, K262, K265, K274, and K294. Particularly, acetylation at K242, K245, and K262 by HAT p300 and CREB binding protein (CBP) is sufficient to decrease the DNA binding affinity and transcriptional activity of FoxO1 [22]. Here, VPA administration decreased the recruitment of FoxO1 to the promoter regions of gluconeogenic genes in vitro, as shown in Figure 3A–D, and in vivo, as shown in Figure 7. FoxO1 binding to the IRE, which is a consensus motif of FoxO1, was also disturbed by pan-HDAC inhibitors such as VPA, SAHA, and TSA, as well as a class I specific inhibitor (MS275) and a class IIa specific inhibitor (MC1568), as shown in Figure 3E. Our results further show that point mutations in three lysine residues (K242, K245, and K262) in the DNA binding domain of FoxO1 confer resistance to HDAC inhibition, which represses the transcriptional activity of wild-type FoxO1, as shown in Figure 4. According to Mihaylova et al., glucagon induces recruitment of HDAC3 and FoxO1 on both the *G6p* and *Pck1* gene promoters, an effect that is abolished when HDAC4/5/7 are depleted [19]. The mechanisms by which HDACs and HATs deacetylate and acetylate nonhistone protein substrates, respectively, suggest that protein acetylation provides a reversible regulatory mechanism similar to that of protein phosphorylation [23]. Interestingly, our results show that the transcriptional activity of FoxO1 is attenuated by MS275 and MC1568, as well as pan-HDAC inhibitors (SAHA, TSA, and VPA). Our data further suggest that HDAC3 and HDAC4 interact with FoxO1, thereby regulating its transcriptional activity; knocked-down of HDAC3 and HDAC4 increased FoxO1 acetylation and suppressed the expression of FoxO1 target genes, as shown in Figure 5. Most reports have shown that HDAC4, a class II HDAC, does not have deacetylase activity, but serves as a scaffold for HDAC3 [24]. The effect of HDAC4 knocked-down and HDAC4 inhibitors on FoxO1 acetylation may be somewhat different [23]. Since HDAC4 acts as a scaffold, HDAC4 knocked-down decreased expression levels of HDAC4, and leads to the inability to recruit HDAC3, resulting in a significant inhibition of FoxO1 transcriptional activity due to the presence of a large amount of acetylated FoxO1. However, when HDAC4 inhibitor (MC1568) was treated, transcriptional activity of FoxO1 may be somewhat higher than that of HDAC4 knocked-down, since HDAC3 can be recruited to some extent. Mammalian class II HDACs are further subdivided into class IIa (HDAC4, 5, 7, and 9) and class IIb (HDAC6 and HDAC10) subtypes. Class IIa HDACs have a very highly conserved deacetylase domain with an evolutionary substitution of a key catalytic tyrosine residue (to histidine) that is conserved in all class I HDACs and might account for their weak enzymatic activity in conventional deacetylation assays [25]. Interestingly, HDAC5 was previously reported to physically interact with FOXO1 in HEK293 cells which is human embryonic kidney cells [18], yet that interaction is not seen in Figure 5A. In the present study, FoxO1 associates with HDAC3 and HDAC4, not HDAC5, in HepG2 cells. Although Mihaylova et al. showed interaction between FOXO1 and HDAC5, they did not show the direct association among FOXO1, HDAC3 or HDAC4 in HEK293. Also, they presented that Forskolin induces FOXO1 and HDAC4, not HDAC5, into the nucleus. On the contrary, our data shows direct interactions among FOXO1, HDAC3, and HDAC4 in HepG2 which is a well-differentiated hepatocellular carcinoma of humans. These data suggest that deacetylation of FOXO1 is regulated on tissue specificity.

The liver of diabetic patients with insulin resistance abnormally overproduces glucose [26]. Glucose homeostasis is controlled by nutrient sensing and hormonal signaling mechanisms that regulate tissue specific rates of glucose production and utilization [27]. Normally, insulin binds to the IR, increases IR tyrosine kinase activity, and phosphorylates IR substrates 1 and 2. Consequently, activation of downstream phosphatidylinositol-3-kinase (PI3K) and protein kinase B/AKT can result in phosphorylation of FoxO, leading to suppression of its nuclear translocation. Activation of AKT increases glycogen synthesis and inhibits gluconeogenesis and glucose production [4,28,29]. However, insulin resistance promotes FoxO1 activation by reducing insulin signaling, resulting in dephosphorylation of the FoxO1 allowing its entry into the nucleus. In addition, glucagon-induced dephosphorylation of class IIa HDACs results in the nuclear translocation and deacetylation of nuclear FoxO1, which enhances FoxO1 DNA-binding activity and its association with gluconeogenic gene promoters [19]. This study shows that blood glucose levels are significantly increased in OLETF rats, an effect that is ameliorated by VPA administration, as shown in Figure 1A,B. Here, decreased blood glucose levels coincided with a decrease in the expression of gluconeogenic genes such as *G6p*, *Fbp*, *Pck1*, and *Pc* via transcriptional repression of FoxO1 resulting from HDAC3 and HDAC4 inactivation, as shown in Figure 1D–G.

FoxO activity is regulated by posttranslational modifications such as phosphorylation, acetylation, ubiquitylation, and sumoylation. These posttranslational modifications affect protein stability, DNA binding affinity, and subcellular localization [30]. Akt phosphorylates FoxO1 at Thr24, Ser253, and Ser316, leading to its nuclear translocation and inactivation, which results in suppression of gluconeogenesis [31]. O-glycosylation of FoxO1 results in the upregulation of *G6P* expression without a change in localization [32]. Deubiquitination of monoubiquitinated FoxO1 via the herpes virus associated ubiquitin specific protease 7 (USP7) decreases FoxO1 recruitment at gluconeogenic gene promoters [33]. Glucose and lipid metabolism are interconnected in many ways; therefore, it is not surprising that diabetic patients present with dyslipidemia characterized by elevated triglycerides, low high-density lipoprotein (HDL), and high low-density lipoprotein (LDL) levels [34]. Microsomal triglyceride transfer protein (MTP) and apolipoprotein C-III (ApoC-III) are two key enzymes involved in lipid metabolism. MTP acts as a transporter for delivering lipids to apoB, catalyzing the rate-limiting step in very low-density lipoprotein-triglyceride (VLDL-TG) production and secretion. ApoC-III acts as an inhibitor of lipoprotein lipase, regulating the rate of VLDL-TG hydrolysis and clearance. FoxO1 acts as a transcriptional enhancer that promotes MTP and ApoC-III production in lipid metabolism [35]. Transgenic mice with a constitutively active FoxO1 allele exhibit hepatic VLDL-TG production, whereas RNAi-mediated FoxO1 deficiency in the liver results in reduced hepatic MTP levels and diminished VLDL production in mice [36].

A recent article reported that VPA induces hepatic steatosis by upregulating clusters of differentiation 36 (CD36) and diacylglycerol acyltransferase 2 (DGAT2) in HepG2 cells and livers of C57B/6J mice; these results are likely attributable to increased peroxisome proliferator-activated receptor gamma or inhibition of the mitogen-activated protein kinase kinase (MEK)-extracellular regulated kinase (ERK) pathway, respectively [37]. However, the authors did not clearly explain the mechanisms, and VPA was administered to healthy C57B/6J mice and HepG2 cells cultured under normal conditions. Experiments with HDAC inhibitors, including VPA, are generally designed to compare pathogenic conditions versus normal conditions. Because the enzyme activity of HDACs is elevated under pathologic conditions, including hypertension [13], HDAC inhibition may attenuate complications. Therefore, VPA exerts different activities under pathologic conditions than under normal conditions.

Sustained hyperglycemia is a major contributor to insulin resistance, which is a hallmark of T2DM [38]. To understand the mechanisms of hyperglycemia in T2DM, we designed a high glucose-induced HepG2 insulin resistance model. Our results show significantly higher expression of *G6P* and *PCK1* in high glucose-induced HepG2 cells than that in cells under normal glucose conditions, as shown in Figure 2. These results might be attributable to reduced phosphorylation of IRS/AKT, leading to FoxO1 nuclear translocation [39,40,41]. Glucose depletion resulted in higher expression of *G6p* and *Pck1* than that observed under normal glucose concentrations. This phenomenon was consistent under fasting conditions, in which activated adenosine monophosphate-activated protein kinase (AMPK) induces class IIa HDAC nuclear translocation, resulting in FoxO1 activation by means of deacetylation [19].

In conclusion, this study demonstrates that repression of transcriptional activity of FoxO1 by HDAC inhibitors ameliorates hyperglycemia in type 2 diabetic rats, as shown in Figure 8B. The downregulation of blood glucose levels coincided with decreased expression of gluconeogenic genes via FoxO1 acetylation. Therefore, HDAC inhibition may be a potential therapeutic strategy for treating diabetes.

## 4. Materials and Methods

### 4.1. Animal Experiments

Male LETO and male OLETF rats aged 14 weeks were purchased from SLC Co. (Shizuoka, Japan). Rats were administered sodium valproate (VPA, 0.71% *w*/*v*) dissolved in drinking water for 20 weeks. VPA was purchased from Sigma-Aldrich (St. Louis, MO, USA). Rats were housed in a room under controlled temperature (23 °C) and 12 h light/12 h dark cycle, with free access to chow and water. For fasting studies (13 and 19 weeks after VPA administration), fasting was started before the dark cycle. After 16 h fasting, the rats were fed food for 10 min. Blood glucose of rats was analyzed with the Accu-Chek Performa kit (Roche-Korea, Seoul, Korea). Liver tissues were frozen in liquid nitrogen and stored at −80 °C until further study. The investigation was conducted in accordance with the National Institutes of Health Guide for the Care and Use of Laboratory Animals and was approved by the Institutional Review Board of Kyungpook National University, and every effort was made to minimize both the number of animals used and their suffering (KNU-2016-0079-2, 20 July 2016).

### 4.2. Periodic Acid–Schiff (PAS) Stain

Liver tissues were fixed in 4% formalin (Sigma, St. Louis, MO, USA) overnight, then dehydrated and embedded in paraffin. The paraffin-embedded samples were sectioned at a thickness of 3 μm. The slides were examined using light microscope images of each group of liver tissue (Nikon Imaging Korea, Seoul, Korea).

### 4.3. Quantitative Real Time PCR (qPCR) and Western Blot Analysis

RNA isolation (QIAzol^®^ Lysis Reagent, Qiagen; Hilden, Germany), cDNA synthesis (RevertAid^TM^ first strand cDNA synthesis kit, Fermentas; Glen Burnie, MD, USA), quantitative PCR with indicated primers, and western blotting were performed as described [23]. Antibodies were purchased from Santa Cruz Biotechnology (acetyl-FoxO1, sc-49437; FoxO1, sc-11350: Santa Cruz, Dallas, TX, USA); Abcam (FoxO1, ab70382; HDAC3, ab32369; HDAC4, ab1437: Cambridge, UK); Sigma (β-actin, AC-15; HA, H 6908; Flag, F4042: St. Louis, MO, USA). Primers for human: *G6P* (NM_000151) Forward: 5′-GTCCACATTGACACCACACC-3′, Reverse: 5′-GAGCCACTTGCTGAGTTTCC-3′; *PCK1* (NM_002591) Forward: 5′-CTGGTTCCGGAAAGACAAAA-3′, Reverse: 5′-AAGTTCAGGGCGTCTTCCTT-3′; *FBP* (NM_000507) Forward: 5′-GCCGTGTTAGACGTCATTCC-3′, Reverse: 5′-CTGGGCAGAGTGCTTCTCAT-3′; *PC* (NM_001040716) Forward: 5′-GCCTGGGAAGGTGATAGACA-3′ Reverse: 5′-ACAGTACCCTCCATGGGTGA-3′ Primers for rat: *G6p* (NM_013098) Forward: 5′-ACCCTGGTAGCCCTGTCTTT-3′, Reverse: 5′-GGGCTTTCTCTTCTGTGTCG-3′; *Pck1* (NM_198780) Forward: 5′-ATACGGTGGGAACTCACTGC-3′, Reverse: 5′-GTTATGCCCAGGATCAGCAT-3′; *Fbp* (NM_012558) Forward: 5′-ACCAGTTATCATGGGGTCCA-3′, Reverse: 5′-ACTCTCGAGCTCTGCTCTGG-3′; *Pc* (NM_012744) Forward: 5′-TCATTAAGGTGGCCAAGGAG-3′, Reverse: 5′-ATGAATCGGACTCCAGCATC-3′.

### 4.4. Immunoprecipitation and Western Blot

The frozen liver tissues were homogenized in radioimmunoprecipitation assay buffer containing protease inhibitors. The lysates were pre-cleared with protein G agarose at 4 °C for 2 h. The supernatants were incubated overnight with 2 μg of FoxO1 antibody (Cell signaling technology, Danvers, MA, USA) at 4 °C. Added protein G agarose to this, and then incubated for 2 h at 4 °C. The immune-complexes were washed three times with lysis buffer (20 mmol/L Tris, 150 mmol/L NaCl, 1% NP-40, 0.5% SDS, 0.1% SDS, and proteinase inhibitor cocktail) and subjected to western blot analysis. For western blot analysis, 1 μg/mL of FoxO1 (Santa Cruz, Dallas, TX, USA) or acetyl lysine (Abcam, Cambridge, UK) antibodies were used.

### 4.5. Cell Culture

HepG2 cells were purchased from the American Type Culture Collection (Manassas, VA, USA) and maintained under 5% CO_2_ at 37 °C in Dulbecco’s modified Eagle’s medium containing 10% fetal bovine serum (Invitrogen, Carlsbad, CA, USA). They were sub-cultured every 3 or 4 days with trypsin (0.25%) and EDTA (0.02%). Cells were treated with several HDAC inhibitors: VPA (Sigma, St. Louis, MO, USA), SAHA (Sigma, St. Louis, MO, USA), and TSA (Sigma, St. Louis, MO, USA); MS275 (Selleckchem, Houston, TX, USA); MC1568 (Santa Cruz, Dallas, TX, USA).

### 4.6. Electrophoretic Mobility Shift Assay

Nuclear extract from HepG2 cells was prepared by nuclear extract-protein extraction reagents (NE-PER Cell Fractionation Kit, Thermo-Fisher Scientific Inc., Rockford, IL, USA) according to the manufacturer’s instructions. Electrophoretic mobility shift assay (EMSA) was performed in a 20 μL binding reaction containing 10 μg of the nuclear extract and biotin-labelled double-stranded oligonucleotides as the probe. For super shift experiments, the nuclear extract was preincubated with anti-FoxO1 antibody (Abcam, Cambridge, UK) for 1 h before incubation with the biotin-labelled probe. DNA protein complexes were separated on 6% nondenaturing polyacrylamide gel in 0.5× TBE for 40 min at 100 V at 4 °C and were transferred to positively charged nylon membrane. The membrane was hybridized with streptavidin-horseradish peroxidase conjugate to detect biotin-labelled DNA by chemiluminescence. 

### 4.7. RNA Interference

For knockdown experiments, small interfering RNA (siRNA) specific for HDAC3, HDAC4, or a negative control (scrambled) (Qiagen, Hilden, Germany) were transfected into HepG2 cells via HiPerFect transfection reagent (Qiagen, Hilden, Germany). Knockdowns were carried out for 72 h. Cells were treated with VPA (10 mmol/L) for 24 h.

### 4.8. Chromatin Immunoprecipitation (ChIP) Assay

ChIP assays were performed according to the manufacturer’s instructions, using the EZ-ChIP™ kit (Upstate Biotechnology, Lake Placid, NY, USA). Tissues were fixed with 1% formaldehyde and washed with phosphate buffered saline containing proteinase inhibitors. After homogenization, tissues were incubated in SDS lysis solution for 10 min on ice. The lysates were sonicated on ice. The lysates were pre-cleared with protein G agarose beads for 2 h. Then, antibodies were added and incubated at 4 °C overnight. To reverse the crosslinking between DNA and chromatin, elutes were incubated at 65 °C for 5 h after the addition of NaCl. DNA was purified with a spin column. Specific promoter DNA was quantified by qPCR. Chromatin was immunoprecipitated with anti-FOXO1 (Abcam, Cambridge, UK), anti-HDAC3 (Abcam, Cambridge, UK), anti-HDAC4 (Abcam, Cambridge, UK), or normal rabbit IgG. Recoveries were calculated as percent of input. Primers for human: *G6P* (NM_000151) Forward: 5′-CCAAGAAGCATGCCAAAGTT-3′, Reverse: 5′ -GCCCTGATCTTTGGACTCAA-3′; *PCK1* (NM_002591) Forward: 5′-TGCCTACTCTATGCCAAGCA-3′, Reverse: 5′-AGTTCATGGGAAGGATGCAC-3′; *FBP* (NM_000507) Forward: 5′-ACTCGAAAAGGGCAGGAGTT-3′, Reverse: 5′-GGTCGCTGACACAGAGTCCT-3′; *PC* (NM_001040716) Forward: 5′-AGCCATATCCACACGGAAAG-3′ Reverse: 5′-CTGCACCTGGGAAAATAAGC-3′ Primers for rat: *G6p* (NM_013098) Forward: 5′-CAGGAGCCACAGTTGAAACA-3′, Reverse: 5′-GCAAAACAGGCACACAAAAA-3′; *Pck1* (NM_198780) Forward: 5′-TACAATCACCCCTCCCTCTG-3′, Reverse: 5′-GCTGCTGGTTGTCAAAACAC-3′; *Fbp* (NM_012558) Forward: 5′-GAGCTCTGGGCTCAAAGAAA-3′, Reverse: 5′-TTCCGCCGAGAAAACTATTG-3′; *Pc* (NM_012744) Forward: 5′-CCTATCACTGGAAGGCAGGA-3′, Reverse: 5′-GCCTCTGAGTGACCTTGGAG-3′.

### 4.9. Luciferase Assay

For the luciferase assay, HepG2 cells were seeded on 12-well plates, cultured overnight, and transfected with vector constructs using Superfect Transfection Reagent (Qiagen, Hilden, Germany), according to the manufacturer’s recommendation. The next day, cells were exposed to 5.5 mmol/L or 30 mmol/L glucose for 24 h with or without pretreatment with VPA (10 mmol/L). The cells were lysed with a lysis buffer and the luciferase activity was analyzed with a Bright-Glo™ Luciferase Assay System kit (Promega, Madison, WI, USA) according to the manufacturer’s instructions. Luminescence was measured with a Veritas™ Microplate Luminometer (Turner Biosystems, Sunnyvale, CA, USA). FoxO1 constructs were purchased from Addgene (Wt-FoxO1, #12142; KA-FoxO1, #66164; KR-FoxO1, 17560; KQ-FoxO1, #1756) (Addgene, Cambridge, MA, USA).

### 4.10. Statistics

Results are expressed as mean ± standard error of mean (SEM). Data were analyzed with the Kruskal–Wallis test or one-way ANOVA followed by post-hoc Tukey’s comparison test; differences were considered significant at *p* < 0.05. Student’s *t*-test was applied for the analysis of the significance of differences between two groups. The procedures were performed using SPSS software (release 19.0, SPSS, Chicago, IL, USA).

## Abbreviations

FoxO1	forkhead box O1
FBP	fructose-1,6-bisphosphatase
G6P	glucose 6-phosphatase
HDACs	histone deacetylases
HDACi	histone deacetylase inhibitors
IR	insulin receptor
IRE	insulin response element
LETO	male Long-Evans Tokushima Otsuka
NFκB	nuclear factor kappa B
OLETF	Otsuka Long-Evans Tokushima Fatty
PGC1α	peroxisome proliferator-activated receptor gamma coactivator 1 alpha
PI3K	phosphatidylinositol-3-kinase
PCK1	phosphoenolpyruvate carboxykinase
PC	pyruvate carboxylase
VPA	sodium valproate
SAHA	suberoylanilide hydroxamic acid
TSA	trichostatin A
T2DM	Type 2 diabetes mellitus
